# Estimating Climate-Sensitive Wildfire Risk and Tree Mortality Models for Use in Broad-Scale U.S. Forest Carbon Projections

**DOI:** 10.3390/f14020302

**Published:** 2023-02-03

**Authors:** Raju Pokharel, Gregory Latta, Sara B. Ohrel

**Affiliations:** 1 Department of Forestry, Michigan State University, East Lansing, MI 48824, USA; 2 Department of Natural Resources and Society, University of Idaho, Moscow, ID 83844, USA; 3 U.S. Environmental Protection Agency, Washington, DC 20004, USA

**Keywords:** climate, carbon sequestration, forests, fuel loading, spatial autoregressive model, tree mortality, wildfire risk

## Abstract

This study utilizes forest inventory and climate attributes as the basis for estimating models of wildfire risk and associated biomass loss (tree mortality) and then demonstrates how they can be applied in calculating CO_2_ emissions related to the incidence of wildfires from U.S. forests. First, we use the full set of over 150,000 FIA plots of national forest inventory and climatic parameters to estimate models of the annual probability of wildfire occurrence and loss of live tree biomass. Then, maps of the spatial allocation of both the model-derived probability of wildfire occurrences and tree mortality are presented at the national level. The probability of wildfire occurrences and tree mortality were defined by a complex non-linear association of climatic conditions and forest ownerships, available aboveground biomass, and the age of the stand. Then, we provide an example of how these models can estimate potential CO_2_ emissions from wildfires by using FIA inventory data. We estimated 6.10, 16.65, 22.75, and 31.01 million metric tons of annual CO_2_ emissions with low, medium, high, and catastrophic combustion rates, respectively, from forests due to wildfire in the continental U.S. The wildfire risk and biomass loss due to tree mortality maps can be used by landowners, managers, public agencies, and other stakeholders in identifying high-risk wildfire zones and the potential CO_2_ emissions. These equations can also help estimate fire risk and associated CO_2_ emissions for future climate conditions to provide insight into climate change-related wildfire occurrences.

## 1. Introduction

**Figure 1.**
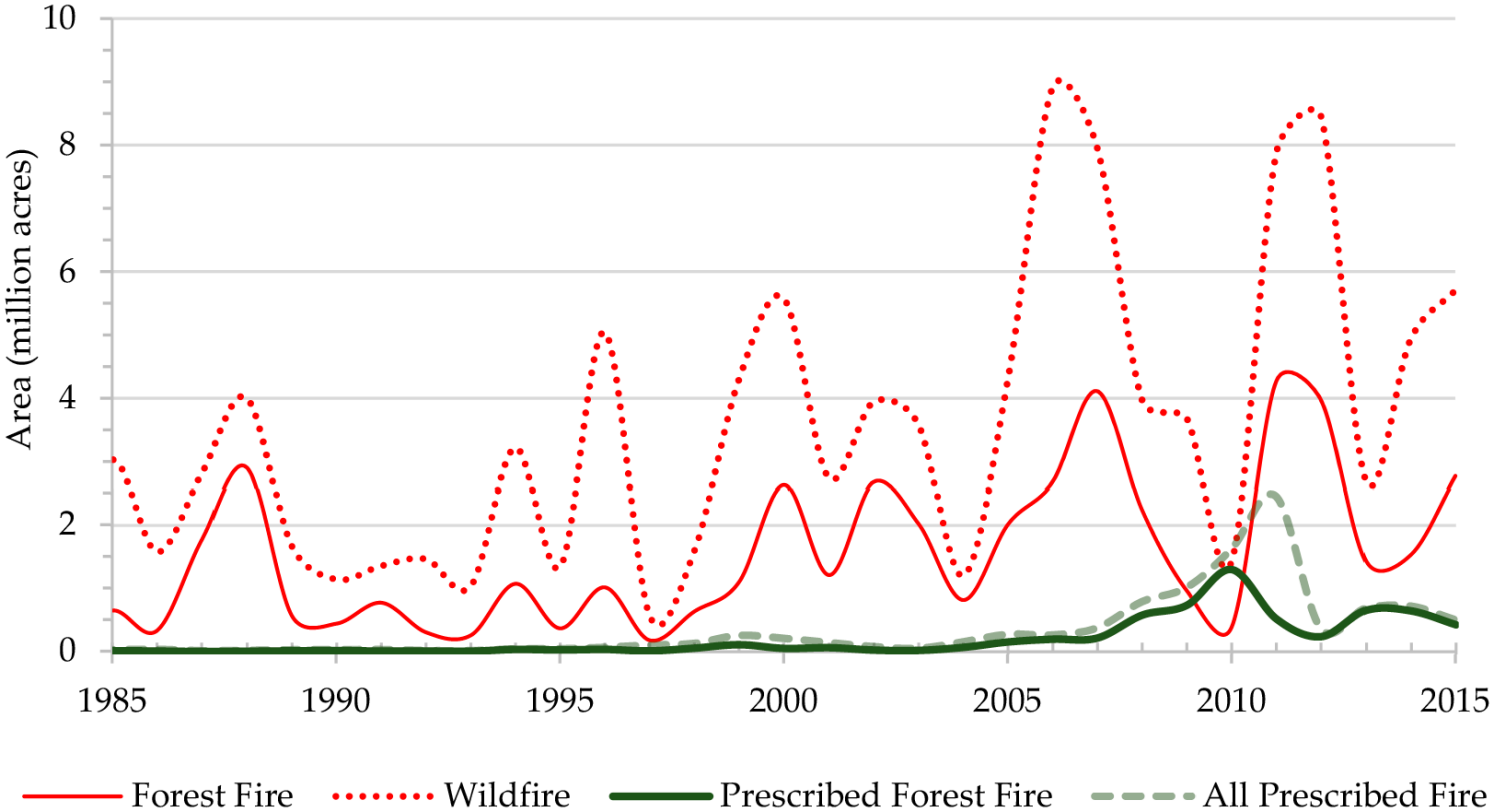
Historical trend of fire in the continental United States. Data from MTBS [3].

Wildfire is an issue that impacts the ecology, economy, safety, and security of ecosystems as well as human welfare. Every year, billions of dollars are spent suppressing and controlling catastrophic wildfires. In 2017, the estimated U.S. economic loss due to wildfire was USD 23 billion, an increase of 12% since 2008 [1]. In the same year, 3400 lives were lost, and 14,670 people were injured by the U.S. wildfires [1]. After the ‘Big Blowup’ (a devastating series of forest fires in 1910 that burned 3 million acres in Montana, Idaho, and Washington), total fire suppression (including a halt of controlled burns) became a major policy focus of the United States Forest Service (USFS). As a result, biomass fuel (i.e., standing and downed deadwood in addition to standing live biomass) accumulated and increased the occurrence and ferocity of wildfires in a range of American wildlands [2,3]. As a result, the number of large fires increased by seven per year and 355 km^2^/year between 1984 and 2011 in the west [4]. Figure 1 shows the area burned between 1985 and 2015 [3]. Though fires are an integral part of natural ecosystems, studies have suggested that human influence, either directly through ignition source [5] or indirectly through management [6] and climate change [7], have altered wildfire dynamics. Consequently, this suppression policy may have altered wildfire behavior to a point where they are no longer a natural process in the U.S. [8,9].

Suppression is not the only factor increasing the number of wildfires in the U.S. In a review of the 17 National Forests management guided by the Northwest Forest Plan over the past 25 years, Spies et al. (2019) noted that a narrow focus on biodiversity conservation strategies could have unintended consequences. Of particular note there is the buildup of biomass. The no or limited harvest application has led forests towards biological maturation (i.e., mainly having older trees, which grow and sequester CO_2_ and store carbon at slower rates than younger trees). Consequently, not all forests domestically are carbon sinks. Some forests, for example, in the northern Rocky Mountains, with old-growth and a lot of dead materials, have already flipped from being net carbon sinks to emitters [10]. Additionally, natural disturbances such as wildfires may have already reduced carbon sequestration rates in the more arid regions of the west [11]. Recent mortality events (e.g., bark beetle kill) related to high carbon density forests of the northwest have increased the fuel load leading to an increased risk of catastrophic forest fires and associated subsequent release of stored carbon [10, 12]. Carbon sequestration rates in the land use, land-use change, and forestry sector decreased by approximately 11.5% between 1990 and 2017 [13]. This decline was primarily a function of declining carbon accumulation in U.S. forests. The historic forest carbon stock trends indicate a declining increase in carbon sequestration in U.S. forests [13]. The United States Mid-Century Strategy, in compliance with the Paris Agreement, identified wildfire reduction as one of the key areas for reducing future U.S. CO_2_ emissions [14]. The 2010 Resource Planning Act Assessment report stated that its GHG projections do not explicitly track CO_2_ emissions from forest fires but that the projected forest carbon stock estimates account for the net effects of fire as a function of mortality [15]. However, changes in forest carbon stocks due to wildfires are highly variable from year to year [13]. The Intergovernmental Panel on Climate Change’s (IPCC) 2006 guidelines for national GHG inventories thus recommends reporting fluxes according to changes within (e.g., natural disturbances such as fire and insect kills) and conversions between all land-use types [16]. Wildfires and related carbon implications cannot be neglected in forest management planning, GHG inventory reporting, and related policy decisions. Therefore, it is critical to developing procedures to estimate wildfire occurrence probabilities and related potential CO_2_ emissions from wildfires.

In estimating CO_2_ emissions from wildfires in U.S. forests, obtaining wildfire information and simulating fire occurrences and fire-related tree mortality is necessary. Wildfire prediction is challenging without the ability to predict ignition and spread mechanisms [17]. Several fire models have been developed, notably models by Preisler et al. [18], Andrews et al. [19], Finney et al. [20], Kerr et al. [21], and Stenzel et al. [22]. Past modeling studies have focused on either a small area or a short time horizon using detailed site characteristics and weather conditions to model fire behavior [19–21]. In some cases, earlier modeling efforts looked more broadly across a region using generalized site characteristics and climatic parameters [23–25]. The highly focused studies cannot be generalized outside of the ecoregions, and the simplification of the generalized regional models may be subject to spatial bias due to misspecification. Although most of the models claim that they can be generalized across the nation, it is not appropriate to use a regional model at a national scale due to varying forest attributes and climatic conditions across different ecoregions in the U.S. Moreover, localized fire behavior models use various data inputs (such as temperature and precipitation, wind speed and direction, soil moisture [26], air pressure, vapor pressure, a surface fuel consumption class, and index of fine dead fuel moisture content [27]), modeled estimates and assumptions (such as fuel strata gap or height to live crown base [27], spread rate [20], combustion factors [22], dryness or Palmer Drought Severity Index (PDSI) [26], and first snowmelt day [8]), and frameworks (ranging from regression analysis [28], Monte Carlo simulations [27], IPCC guidelines [16], to machine learning [29,30]) and may provide inconsistent estimates at the national scale across different regions. Moreover, these fire behavior models estimate fire risk, intensity, and spread based on a complex set of parameters such as wind speed and directions, vapor pressure, fuel loading, moisture content, etc., that are hard to predict in the future. A compelling motivation of this study is to produce a set of equations to estimate future wildfire risk and associated tree mortality based on climate and forest conditions such that national risk assessment and CO_2_ emissions due to wildfires over time can be simulated using forest sector models. Therefore, we designed this study to build and validate wildfire risk and associated tree mortality equations that can consistently estimate risk and mortality for forestland of the continental U.S., which can be readily and efficiently used in the various modeling approaches and risk assessments.

The objective of this study is to derive a climate-sensitive model that can be used to generate consistent estimates of wildfire probability and fire-related tree mortality across the continental U.S. We perform this through the development of two independent equations using climatic parameters and forest attributes: ownership in the case of wildfire probability and bring in fuel loading through aboveground biomass to determine subsequent tree mortality. We then demonstrate how these equations can be used to generate estimates of wildfire-caused U.S. forest carbon emissions or generate maps at the national scale. These equations are then used to calculate area burned, live biomass lost due to wildfire, and CO_2_ emitted to cross-validate as well as to demonstrate the applicability of these equations at the national scale.

## 2. Materials and Methods

### 2.1. Data

Forest inventory data were obtained from FIA DataMart of the USDA Forest Inventory and Analysis (FIA) National Program [31]. FIA is a continuing endeavor mandated by the Forest and Rangeland Renewable Resource Planning Act of 1974 and is in charge of assessing the country’s forest resources, including condition, volume, growth, and ownership changes, and has established a grid of permanent inventory plots across the country [32,33]. The 1998 Farm Bill required FIA to collect data on plots annually within each state. The enhanced FIA program consists of three phases. We used phase 2 data information, consisting of one field sample site for every 6000 acres. Different attributes such as forest type, site, tree species, tree size, and conditions of forest land are available.

**Figure 2.**
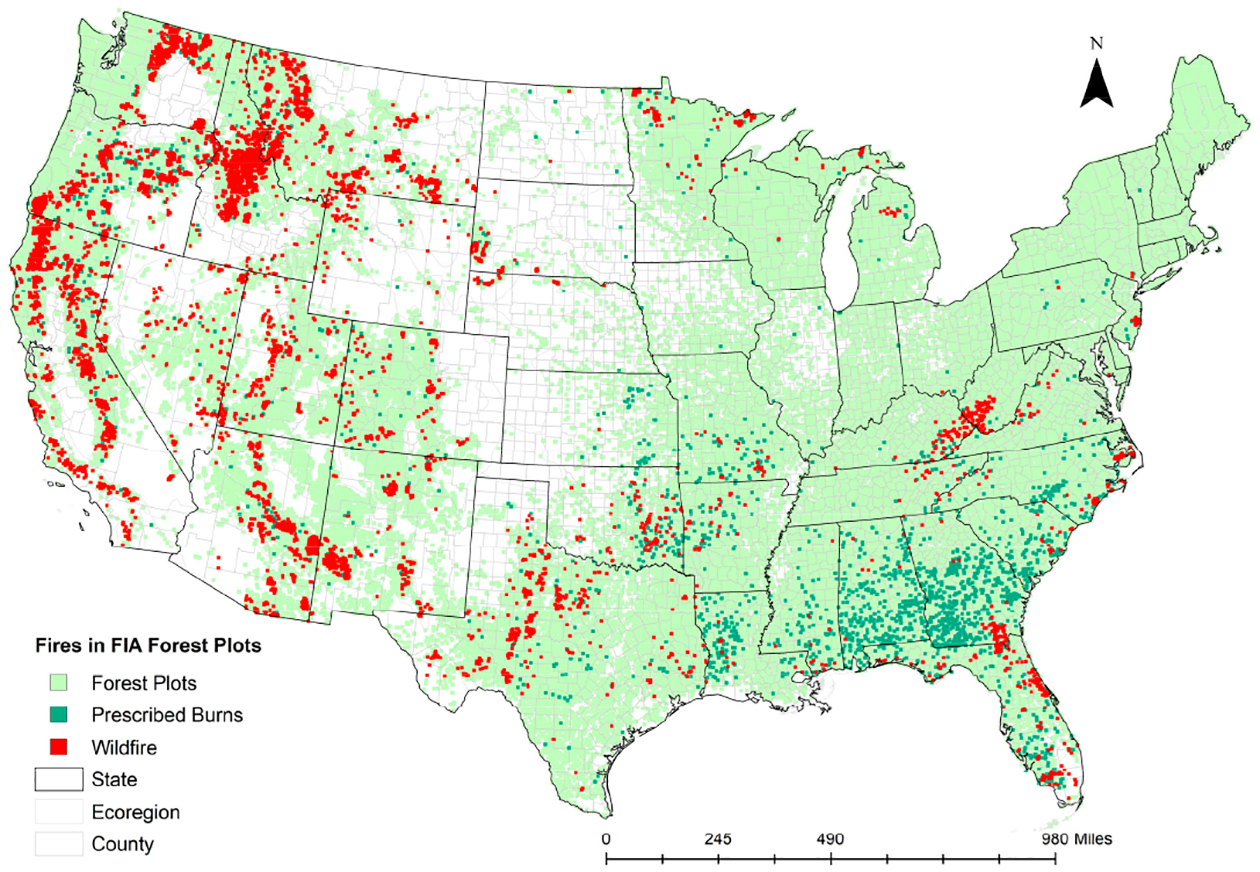
FIA forested plots with historic wildfires and prescribed burns between 2000 and 2015 [3,26].

The condition information of the plot’s location was recorded. Conditions are defined by land use changes or vegetation changes that occur along more-or-less distinct boundaries. USFS remeasures FIA plots at regular intervals, approximately five years in the southern and seven to ten years in the northern United States. We combined tree-level information such as diameter, height, volume, biomass, and species with plot or condition class-level information on location, aspect, slope, elevation, ownership, and disturbances due to fires between 2000 and 2015. Finally, after identifying plots that experienced fires between 2000 and 2015 (Figure 2) and calculating wildfire probabilities, the data were compared and overlaid with the Monitoring Trends in Burn Severity (MTBS) fire perimeters [3]. The prescribed burns reported in MTBS allowed us to eliminate corresponding FIA plots from our wildfire data set. Finally, an eco-province was assigned for each plot [34].

On average, approximately 7% (SD = 25%) of the FIA forested plots experienced wildfires between 2000 and 2015 (Table 1). When there was a wildfire in any plot, 23% (SD = 36%) of the live tree biomass was lost (dead or burned). The average aboveground biomass was 26,824 metric tons per hectare (SD = 30,800). On average, the stands were 65 years old (SD = 55.40), and 25% (SD = 44%) of these forested plots were federally owned. The west experienced most of the wildfires compared with other regions, especially in the inland of the Pacific Northwest (Washington and Oregon), California, Idaho, and Montana between 2000 and 2015 (Figure 2). Prescribed burns were popular in the southeastern states as a forest management tool and became a common treatment after 2000 (Figures 1 and 2). There were prescribed burns in the west and other regions also, but the practice was not as widespread as in the southeast.

The climate data were obtained from the Parameter-elevation Relationships on Independent Slopes Model (PRISM) Climate Group [35,36]. Monthly averages of precipitation, maximum temperature, and minimum temperatures were obtained for 30-year normal (an average over a recent 30-year period, 1985–2015) for the U.S. from a raster of 0.16 square km (800 m × 800 m) pixel size. Wildfires are seasonal phenomena, most often occurring in the dry and hot climate typical of summer and early autumn. Therefore, we defined the fire season as the period between July and October, and climate variables were averaged over these four months. However, the climatic conditions in spring also affect the growth of the trees, hence the fuel conditions (biomass) in the forest. A wet spring with a high temperature corresponds with higher biomass [37], hence increasing the fuel load on the landscape. Moreover, the water cycle and water availability in the fire season depend on the climatic conditions during the spring season [38]. We defined the spring season as the period between February and April, and climate variables were averaged over these three months. During the fire season, the average precipitation was 82.00 mm, and the maximum temperature was 25.09 °C for the continental U.S. (Table 1). During the spring season, the average precipitation was 87.28 mm, and the average monthly maximum temperature was 12.08 °C. Monthly average evapotranspiration using 30-year normal was calculated using a modified Hargreaves equation as specified in Equation (1) [39].

(1) $$ET=\left(0.0135\frac{{\text{Temp}}_{max}-{\text{Temp}}_{min}}{2}+17.78\right)\;R\left(\frac{a}{595.5-0.55\frac{{\text{Temp}}_{max}-{\text{Temp}}_{min}}{2}}\right)$$

where ET is the potential daily evapotranspiration in millimeters per day, $Temp_{max}$ is the monthly maximum temperature in degrees Celsius, $Temp_{\text{min}}$ is the monthly minimum temperature in °C, R is the incident solar radiation calculated as a function of the slope, aspect, and elevation of the plot, solar input (direct radiation hitting the ground), and transmittance in MJ/m^2^/day, and a is a constant of 238.80 when R is expressed as MJ/m^2^/day. The average evapotranspiration was 405 mm (SD = 69) during the fire season and 220 mm (SD = 72) during the spring season.

**Figure 3.**
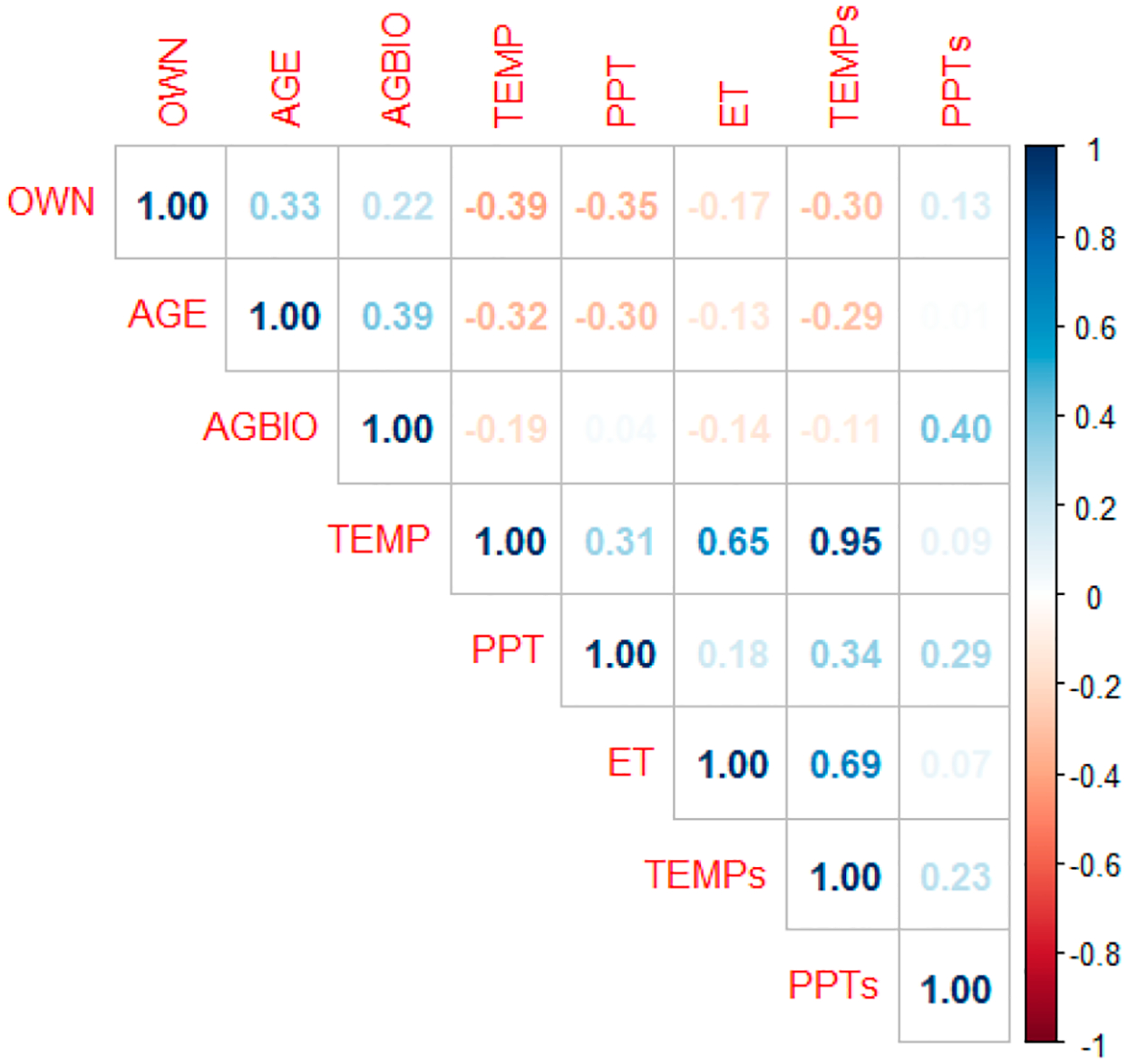
Correlation between the climate and other variables.

The climate variables are correlated in space and time; therefore, we calculate the Pearson Correlation Coefficient [40] using Equation (2) to understand the interrelation between the variables used in this study.

(2) $$\rho_{x,y}=\frac{cov(x,y)}{\sigma_{x}\sigma_{y}}$$

where $\rho_{x,y}$ is the Pearson correlation coefficient, cov is the covariance, $\sigma_{x}$ is the standard deviation of x-climate and other variables used in this study, $\sigma_{y}$ is the standard deviation of y-climate and other variables used in this study. Figure 3 shows the correlation between different variables.

Maximum temperature in summer is highly correlated with spring temperatures $\left(\rho_{x,y}=0.95\right)$, summer evapotranspiration (ρ=0.65), and moderately correlated with summer precipitation $\left(\rho_{x,y}=0.31\right)$. Summer precipitation was moderately correlated with spring temperatures $\left(\rho_{x,y}=0.34\right)$ and precipitation $\left(\rho_{x,y}=0.29\right)$. Summer evapotranspiration was strongly correlated with spring temperatures $\left(\rho_{x,y}=0.69\right)$. Aboveground biomass has a positive correlation with spring precipitation $\left(\rho_{x,y}=0.40\right)$ and a weak or no correlation was observed with other climate variables. Stand age has a negative correlation with summer temperature $\left(\rho_{x,y}=-0.32\right)$ and precipitation $\left(\rho_{x,y}=-0.30\right)$, spring temperature $\left(\rho_{x,y}=0.29\right)$ and positive correlation with aboveground biomass $\left(\rho_{x,y}=0.39\right)$. Public forest ownership is negatively correlated with spring temperature $\left(\rho_{x,y}=-0.39\right)$, and precipitation $\left(\rho_{x,y}=-0.35\right)$, spring temperature $\left(\rho_{x,y}=-0.30\right)$, and positively correlated with the age of the stand $\left(\rho_{x,y}=0.33\right)$.

### 2.2. Variable Descriptions and Model Specification

To model wildfire risks and associated tree mortality, it is crucial to understand the conditions or factors that help trigger wildfires [41]. Wildfire incidence is a combined effect of human, climate, forest, and fuel-loading conditions on the landscape. Humans start most wildfires. However, the propagation and ferocity depend on climatic conditions and the fuel available in the landscape. Fuel for wildfire is biomass, both dead and alive, readily available to ignite and keep the fire alive. Climate conditions serve as critical thresholds, influencing wildfire size and spread at different spatial scales [42]. Historically, the U.S. west has experienced large fires due in part to its dry climate [2,43]. Dryness, coupled with higher volumes of fuel (biomass), can increase the intensity of fire across a landscape [44]. The interaction between temperature, moisture conditions, and the land’s topography dictates the area’s dryness [45].

We used climate parameters (temperature and moisture conditions), forest biomass, and forest conditions to estimate the annual probability of wildfire occurrences and live tree mortality due to tree mortality. In Equation (2), FIRE is a binary variable, with a ‘1’ indicating a wildfire recorded on an FIA plot and a ‘0’ otherwise. Previous studies used climate variables related to fuel conditions such as wind speed, precipitation, maximum and minimum temperatures, soil moisture, and the Palmer Drought Severity Index (PDSI) to model fire behavior [8,26,37,42,46–48]. Most studies concluded that high temperature and low moisture conditions are hazardous and aid in wildfire risks. To keep the model parsimonious and simple, after several iterations of regression of various climate variables on our fire and mortality model, we decided to use maximum temperature, precipitation, and evapotranspiration as climate variables. Precipitation only accounts for the water available but not its retention in the landscape; therefore, we included an average of the monthly evapotranspiration to represent moisture-related impacts. Use of these variables would allow us to use these models for wildfire and mortality predictions in the future, using various climate models to project the impact of climate change in wildfire probabilities. Since not all seasons have fire risks, we used the average maximum monthly temperature, precipitation, and evapotranspiration for the fire season or summer (TEMP, PPT, ET) and spring season (TEMPs, PPTs, ETs) to estimate wildfire occurrence and tree mortality. Further, ownership (OWN) impacts the management regime of a forest, thus affecting fuel buildup. Starrs et al. [47] built on numerous other studies and concluded that ownership, firefighting, and reserve funds were significant in determining wildfire probabilities, where federally owned forests were more likely to burn. Privately owned forests are regularly thinned and prescribe-burned in many parts of the U.S., especially in the south, to maintain forest health and increase yield. Public forests, mostly federally owned, are typically managed less aggressively compared with private forests for different reasons, including but not limited to habitat preservation and other non-market benefits or low human or financial resources. We initially used slope, aspect, and elevation of FIA plots in the model but decided to drop them since climate variables were highly correlated with them [26]. Moreover, these variables were insignificant and increased RMSE and decreased FI. The forest management practices affect fuel conditions, tree spacing, and related fire hazards. Thus, the annual probability of wildfire occurrence in the i^th^ plot $\left(p_{i}\right)$ was estimated as a function of climate variables and forest ownership (Equation (3)).


(3) $$\text{FIRE}\left(p_{i}\right)=f\;(\text{PPT},\text{TEMP},\text{ET},\text{PPTs},\text{TEMPs},\text{ETs},\text{OWN})$$

Tree mortality in the event of a fire is highly dependent on the climatic and fuel (biomass) conditions in the forests [49]. A plot with higher live tree biomass loses more biomass compared with a plot with less biomass on it [50]. Moreover, including forest age in quantifying spatial–temporal variation using empirical approaches is meaningful and important [51]. Since young trees are more vulnerable to fire mortality and can act as ladder fuel to ignite the crown, the proportion of live biomass loss is higher in young stands in the event of fire [52]. Our analysis also showed aboveground biomass has a positive correlation with spring precipitation ($\rho_{x,y}=0.40$, Figure 3). We included both total aboveground biomass in the plot (AGBIO) and stand age (AGE) together to separate the effect of additional biomass due to increasing girth of trees due to age or increasing number of smaller trees or understory growth. Next, TREELOSS was calculated as a proportion or ratio of the biomass of dead and missing trees to the total aboveground standing biomass in a plot. To calculate the biomass of dead and missing trees, we identified all FIA plots with reported fires between 2000 and 2015. Then, we used condition class and indexing in remeasurements performed at FIA plots to identify dead or missing trees between remeasurements on these plots and estimated the biomass of those trees. Remeasurements were only tracked on plots with reported fires to determine and identify missing, dead, or alive trees. If there were more than two remeasurements in a plot, we used the recent two measurements to estimate TREELOSS. Equation (4) is used to estimate the annual probability of tree mortality (TREELOSS) in the event of wildfire to the total aboveground biomass in trees in the i^th^ $\text{plot}\left(m_{i}\right)$.


(4) $$\text{TREELOSS}\left(m_{i}\right)=f\;(\text{PPT},\text{TEMP},\text{ET},\text{PPTs},\text{TEMPs},\text{ETs},\text{OWN},\text{AGBIO},\text{AGE})$$

### 2.3. Model Fitting and Validation

For a start, equations to estimate the annual probability of wildfire occurrence (Equation (3)) and tree mortality (Equation (4)) were fitted using an ordinary least square (OLS) regression, where FIRE and TREELOSS were a function of climatic and forest attributes with quadratic and all interaction terms. Since the fire events in the FIA plots are highly correlated in space with each other in proximity, a spatial autocorrelation test, as specified in Equation (5) on regression residuals (error), was administered using Moran’s I test [53]. Spatial autocorrelation is a measure of the degree of spatial dependence of FIRE or TREELOSS between FIA forest plots at two different locations or points in space.

(5) $$\text{Moran’s}\;I=\frac{N}{W}\frac{\sum_{i=1}^{n}\sum_{j=1}^{n}w_{ij}\left(x_{i}-\overset{‾}{x}\right)\left(x_{j}-\overset{‾}{x}\right)}{\sum_{i=1}^{n}{\left(x_{i}-\overset{‾}{x}\right)}^{2}}$$

where N is the number of spatial units indexed, such as plots, W is the aggregate of all the spatial weights, and $w_{ij}$ is the spatial weight of the i^th^ plot. We used the inverse of the squared distance (1/d^2^) to calculate $w_{ij}$ between plot i and j.

As expected, the OLS regression could not eliminate spatial correlations. Then, we attempted logit regression following Preisler et al. [18], which did not work either. Moving forward, we ran 37 different OLS and logit regressions to model Equations (3) and (4) for 37 U.S. eco provinces, individually and simultaneously following a similar approach to Littell et al. [54]. None of these approaches were able to eliminate spatial autocorrelation. After several other regressions, the spatial autocorrection regression (SAR) satisfied Gauss Markov’s condition for unbiased and consistent estimators to eliminate spatial autocorrelation. SAR is a non-linear regression that accounts for and corrects the autocorrelation due to the spatial proximity using the spatial lags on all variables [55].

(6) $$y_{s}=\alpha +\rho y_{s-1}+\beta_{i}\left(X_{i,s}-\rho X_{i,s-1}\right)+\delta_{i}Z_{i}+\varepsilon$$

where $y_{s}$ is the dependent variable (FIRE or TREELOSS), α is the intercept (a constant), ρ is the spatial autoregressive coefficient, $X_{i,s}$ and $Z_{i}$ are the independent variables, $X_{i,s-1}$ is a spatial lag of $X_{i,s},\beta_{i}$ and $\delta_{i}$ represent parameter estimates, and ε is the SAR error. The spatial lags, $X_{i,s-1}$, were calculated using spatial weights matrix estimated using inverse squared distance function on the nearest neighbors, i.e., FIA forest plots $\left(X_{i,s}/{\text{d}}^{2}\right)$. d is the average distance between the i^th^ plot and all other plots within a radius of 660 km (3-degree geo-coordinate radius) from $X_{i,s}$. Plots that are 660 km or further away are assumed to exhibit no spatial autocorrelation on each other. The effect of spatial autocorrelation in $X_{i,s}$ is corrected by variable transformation as $\left(X_{i,s}-\rho X_{i,s-1}\right)$. Backward elimination was administered to eliminate interaction terms to achieve the lowest root mean squared error (RMSE) and highest fit index (FT). We include the variables that were not significant in the model but were included in the interaction if they improve the FT and reduce RMSE. The fit index in non-linear regression is similar to the R-squared in OLS regressions and measures how well-observed data fit the probability distribution of SAR regression.

The annual probability of wildfire occurrence $\left(p_{i}\right)$ data were aggregated at the county-level ecoregions. To estimate the annual probability of tree mortality ${(m}_{i})$, Gauss Markov’s conditions eliminating spatial autocorrelation were satisfied at FIA forested plot data without the data aggregation. The annual probability of wildfire occurrence $\left(p_{i}\right)$ for an FIA forested plot could only eliminate the spatial autocorrelation when data were aggregated at the county-level ecoregions (Moran’s I=0.13, p-value = 0.19), but could not be achieved at plot level (Moran’s I = 0.72, p-value = 0.0001). After exhausting the possibility of eliminating spatial autocorrelation using an FIA plot as a unit of measurement, we decided to aggregate data to a county-level ecoregion, assuming consistent climatic and forest conditions in these smaller ecoregions of an eco-province divided by counties, combining the approaches used by Littell et al. (2009) and Latta et al. [56]. We tried lessening the impact due to data aggregation by avoiding data aggregation either only into counties recognizing that a county is merely a political boundary or data aggregation into large eco-provinces recognizing the sheer size of the ecoregions that does not accurately reflect underlying climatic and ecological differences. This approach converted 150,350 FIA forested plots into 3873 aggregate forest data points. For example, Latah County in Idaho has 51 FIA plots across two ecoregions: Great Plains-Palouse Dry Steppe (Palouse) and Northern Rocky Mountain Forest-Steppe-Coniferous Forest-Alpine Meadow (Mountain). Instead of aggregating data into a single data point for Latah, 17 plots in the Palouse and 34 plots in the Mountain were averaged to obtain two data points that assume the same climatic and ecological conditions. The overall FT improved to 56% compared with the SAR fitted for plot-level data (30%). The annual probability of tree mortality due to wildfire for an FIA forested plot was estimated using SAR in Equation (5) without an issue of spatial autocorrelation (Moran’s I = 0.09, p=0.84) with an FT of 57%.

The outcomes of SAR estimating the annual probability of wildfire occurrence and tree mortality were validated using ArcGIS overlaying the estimated probabilities with the perimeter and the point data of historic fires in the U.S. provided by MTBS [3] and Esri [57]. This study’s areas with higher estimated wildfire probabilities corresponded with the perimeters and points that historically had frequent and large fires.

Finally, we propose two statistical models fire risk and TreeBio-Loss equations, as specified in Equations (7) and (8) to estimate the annual probability of wildfire occurrence and tree mortality in an FIA forested plot in the event of a wildfire in the continental U.S.

Fire risk equation:

(7) $$\hat{p}=\hat{\alpha }+\rho \;{\text{FIRE}}_{s-1}+{\hat{\beta }}_{1}\;\left(PPT_{s}-\rho \;PPT_{s-1}\right)+{\hat{\beta }}_{2}\;\left(TEMP_{s}-\rho \;TEMP_{s-1}\right)+{\hat{\beta }}_{3}\;\left(ET_{s}-\rho \;ET_{s-1}\right)+{\hat{\beta }}_{4}\;\left(PPTs_{s}-\rho \;PPTs_{s-1}\right)+{\hat{\beta }}_{5}\;\left({\text{TEMPs}}_{s}-\rho \;\text{TEMP}s_{s-1}\right)+{\hat{\beta }}_{6}\;\left(ETs_{s}-\rho \;ETs_{s-1}\right)+{\hat{\beta }}_{7}\;\left(PP{T^{2}}_{s}-\rho \;PP{T^{2}}_{s-1}\right)+{\hat{\beta }}_{8}\;\left(TEM{P^{2}}_{s}-\rho \;TEM{P^{2}}_{s-1}\right)+{\hat{\beta }}_{9}\;\left(E{T^{2}}_{s}-\rho \;E{T^{2}}_{s-1}\right)+{\hat{\beta }}_{10}\;\left({{\text{PPTs}}^{2}}_{s}-\rho \;PPT{s^{2}}_{s-1}\right)+{\hat{\beta }}_{11}\;\left({{\text{TEMPs}}^{2}}_{s}-\rho \;{{\text{TEMPs}}^{2}}_{s-1}\right)+{\hat{\beta }}_{12}\;\left(ET{s^{2}}_{s}-\rho \;ET{s^{2}}_{s-1}\right)+{\hat{\beta }}_{13}\;\left({\left(PPT*\text{TEMP}\right)}_{s}-\rho \;{\left(PPT*\text{TEMP}\right)}_{s-1}\right)+{\hat{\beta }}_{14}\;\left({\left(PPT*ET\right)}_{s}-\rho \;{\left(PPT*ET\right)}_{s-1}\right)+{\hat{\beta }}_{15}\left({\left(PPT*\text{PPTs}\right)}_{s}-\rho \;{\left(PPT*\text{PPTs}\right)}_{s-1}\right)+{\hat{\beta }}_{16}\;\left({\left(\text{TEMP}*\text{TEMP}s\right)}_{s}-\rho \;{\left(\text{TEMP}*\text{TEMPs}\right)}_{s-1}\right)+{\hat{\beta }}_{17}\;\left({\left(\text{TEMP}*ET\right)}_{s}-\rho \;{\left(\text{TEMP}*ET\right)}_{s-1}\right)+{\hat{\beta }}_{18}\;\left({\left(ET*ETs\right)}_{s}-\rho \;{\left(ET*ETs\right)}_{s-1}\right)+{\hat{\delta }}_{1}\;\text{OWN}$$

TreeBio – Loss equation:

(8) $$\hat{m}=\hat{\alpha }+\rho \;{\text{BIOLOSS}}_{s-1}+{\hat{\beta }}_{1}\;\left(PPT_{s}-\rho \;PPT_{s-1}\right)+{\hat{\beta }}_{2}\;\left(TEMP_{s}-\rho \;TEMP_{s-1}\right)+{\hat{\beta }}_{3}\;\left(ET_{s}-\rho \;ET_{s-1}\right)+{\hat{\beta }}_{4}\;\left(PPTs_{s}-\rho \;PPTs_{s-1}\right)+{\hat{\beta }}_{5}\;\left(\text{TEMP}s_{s}-\rho \;\text{TEMP}s_{s-1}\right)+{\hat{\beta }}_{6}\;\left(ETs_{s}-\rho \;ETs_{s-1}\right)+{\hat{\beta }}_{7}\;\left(PP{T^{2}}_{s}-\rho \;PP{T^{2}}_{s-1}\right)+{\hat{\beta }}_{8}\;\left(TEM{P^{2}}_{s}-\rho \;TEM{P^{2}}_{s-1}\right)+{\hat{\beta }}_{9}\;\left(E{T^{2}}_{s}-\rho \;E{T^{2}}_{s-1}\right)+{\hat{\beta }}_{10}\;\left({{\text{PPTs}}^{2}}_{s}-\rho \;{{\text{PPTs}}^{2}}_{s-1}\right)+{\hat{\beta }}_{11}\;\left({{\text{TEMPs}}^{2}}_{s}-\rho \;{{\text{TEMPs}}^{2}}_{s-1}\right)+{\hat{\beta }}_{12}\;\left(ET{s^{2}}_{s}-\rho \;ET{s^{2}}_{s-1}\right)+{\hat{\beta }}_{13}\;\left({\left(PPT*\text{TEMP}\right)}_{s}-\rho \;{\left(PPT*\text{TEMP}\right)}_{s-1}\right)+{\hat{\beta }}_{14}\;\left({\left(PPT*ET\right)}_{s}-\rho \;{\left(PPT*ET\right)}_{s-1}\right)+{\hat{\beta }}_{17}\;\left({\left(\text{TEMP}*ET\right)}_{s}-\rho \;{\left(\text{TEMP}*ET\right)}_{s-1}\right)+{\hat{\beta }}_{18}\;\left({\left(ET*ETs\right)}_{s}-\rho \;{\left(ET*ETs\right)}_{s-1}\right)+{\hat{\beta }}_{15}\left({\left(PPT*\text{PPTs}\right)}_{s}-\rho \;{\left(PPT*\text{PPTs}\right)}_{s-1}\right)+{\hat{\beta }}_{16}\;\left({\left(\text{TEMP}*\text{TEMP}s\right)}_{s}-\rho \;{\left(\text{TEMP}*\text{TEMP}s\right)}_{s-1}\right)+{\hat{\delta }}_{1}\;OWN+{\hat{\delta }}_{2}\;AGE+{\hat{\delta }}_{3}\;\text{AGBIO}$$

### 2.4. Combustion Emissions Due to the Wildfires in the United States

Using the estimated annual probability of wildfire occurrences from the fire risk equation, the total area burned (A) in the continental U.S. can be calculated as specified in Equation (9).

(9) $$A=\sum \left(a\;p_{i}\right)$$

where a is the area of a plot and $p_{i}$ is the annual probability of wildfire occurrence from fire risk equation on i^th^ plot or c^th^ ecoregion within a county. The total aboveground biomass (B) in dead trees due to wildfires in the continental U.S. is calculated as specified in Equation (10).

(10) $$B=\sum \left(a\;p_{i}\;m_{i}\;B_{b}+a\;p_{i}\;m_{i}\;B_{o}\right)$$

where $m_{i}$ is the annual probability of aboveground tree mortality on i^th^ plot estimated from the TreeBio-Loss equation, $B_{b}$ and $B_{o}$ are the total bole and stump biomass and tops, branches, and sapling biomass, respectively, in the plot. Finally, the total carbon dioxide emission (C) is calculated using Equation (11).

(11) $$C=0.5\;M\sum \left(a\;p_{i}\;m_{i}\;B_{b}\;f_{b}+a\;p_{i}\;m_{i}\;B_{o}\;f_{o}\right)$$

where 0.5 corresponds with the assumption that 50% of the dry biomass in a tree is carbon, M is the molecular weight of the carbon dioxide, $f_{b}$ and $f_{o}$ are the combustion emission conversion factors that represents the proportion of bole and stump biomass and biomass and tops, branches, and sapling biomass, respectively, in the event of wildfire at a plot. The combustion conversion of biomass into CO_2_ emissions depends on the intensity of the fire. Stenzel et al. [22] argued the model predictions on CO_2_ emissions from wildfire combustion is overestimated. His study of two wildfires in California resulted in a decrease in estimated emissions from 87% to as low as 10%. Stenzel et al. [22] and Loehman et al. [58] used combustion conversion factors between 5% and 80% of bole biomass and 40%−100% of other tree biomass with low, medium, and high intensity of fires. Similar biomass–carbon conversion is widely used in forest sector modeling [56,59–62]. With the lack of burn intensity data in the FIA, it was impossible to assign combustion factors to each plot or fire event; therefore, we modeled different emission scenarios with varying combustion conversion rates for the bole and other structures of the trees.

We present 12 scenarios with four combustion conversion rates and three different methods of estimating the annual probability of wildfire occurrences and tree mortality in Table 2. The first part of the scenario name indicates the methods employed in estimating probabilities of wildfire and tree mortality, and the second part represents the combustion conversion rates. For example, scenario *AVG_low* estimates CO_2_ emissions using average fire event $\left(p_{i}=\overset{‾}{p}\right)$ and average tree mortality over the past ten years $\left(m_{i}=\overset{‾}{m}\right)$ and low combustion conversion rates $\left(f_{b}=0.05,f_{o}=0.5\right)$. Scenario names starting with AVG estimate CO_2_ emissions using average fire event $\left(p_{i}=\overset{‾}{p}\right)$ and average tree mortality of the past ten years (2005–2015, $\left.m_{i}=\overset{‾}{m}\right)$ A county-level annual wildfire probability was used since fire events are not the same across the U.S., and previous modeling studies [60, 62, 63] have also used county averages. Scenario names starting with *ECP* and *PLT* estimate CO_2_ emissions using this study’s annual probabilities of wildfire risk and tree mortality equations. Both scenarios used plot-level probability estimates for TreeBio-Loss $\left(m_{i}={\overset{ˆ}{m}}_{i}\right)$ but different probability estimates for wildfire risk. Scenario names starting with *ECP* used aggregated estimate of wildfire risk at an ecoregion in a county $\left(p_{i}={\overset{ˆ}{p}}_{c}\right)$ whereas scenario names starting with PLT used annual probabilities estimates of wildfire risk for each FIA plot $\left(p_{i}={\overset{ˆ}{p}}_{i}\right)$. The second part or the suffixes on the AVG, ECP, and PLT scenarios reflect biomass to carbon conversions rates. We used four different combustion conversion factors based on Stenzel et al. [22] and Loehman et al. [58] to estimate the potential biomass conversion to CO_2_ emissions. A scenario with the suffix ‘_*low*’ represents low combustion conversions $\left(f_{b}=0.05,f_{o}=0.5\right)$ with only about 5% conversion of bole and stump biomass and 50% conversion of tops, branches, and sapling biomass. Stenzel et al. [22] reported that at least 5% of biomass is converted to CO_2_ emissions in any fire event and reported that on the lower end of moderate-severity fires, models use a 30% combustion conversion of bole and stump biomass and 80% conversion of tops, branches, and sapling biomass. A scenario with the suffix’_*med*’ represents moderate combustion conversions $\left(f_{b}=0.3,f_{o}=0.8\right)$. On the upper range for similar fire intensity, combustion conversion can be 46% on bole and stump biomass and 92% conversion on tops, branches, and sapling biomass. Scenario with suffix ‘_*high*’ represents the high combustion conversions $\left(f_{b}=0.42,f_{o}=0.92\right)$. Combustion conversions in various models range 30%−80% on bole and 80%−100% on branches and foliage in high-severity fires. Recent studies have [58,64] indicated increasing combustion conversions; therefore, we modelled emission form high with suffix ‘_*cats*’ representing catastrophic combustion conversions $\left(f_{b}=0.8,f_{o}=1\right)$ with 80% conversion on the bole and stump biomass and 100% on tops, branches, and sapling biomass where most of the carbon sequestered in a plot is released.

## 3. Results

### 3.1. Annual Probabilities of Wildfire Occurrence from Fire Risk Equation

The annual wildfire probability of wildfire was positively associated with forest land ownership, where federal lands were more likely to have wildfires than private and state forest lands. The annual wildfire probability for fire risk was negatively associated with the summer precipitation and evapotranspiration, and spring temperature (p<0.01) and positively associated with summer temperature (p<0.01) (Table 3). Spring precipitation and evapotranspiration were not significant in explaining wildfire probabilities. The negative impact of precipitation and evapotranspiration indicates higher wildfire risk in the areas with lower precipitation and evapotranspiration during the fire season, indicating that dry areas or areas with lower moisture content are more prone to wildfires. As expected, there was a positive effect of summer temperature on wildfire probabilities, indicating hotter summers increase the risk of wildfires. The negative coefficient of spring temperature indicates that hotter springs have a lower probability of wildfires in the summer.

All quadratic relationships were significant (p<0.01) in the model, indicating that the marginal effect of climatic variables was non-linear, where the marginal effect increased with increasing summer precipitation and spring temperature while it decreased with increasing summer temperature and evapotranspiration. Hence, the wildfire probabilities increased with higher summer temperatures but at a decreasing rate. The wildfire probabilities decreased with higher precipitation but at an increasing rate.

Although spring precipitation and evapotranspiration were not directly associated with wildfire probability (p>0.10), the model’s interaction terms between spring precipitation, temperature, and evapotranspiration with summer climate conditions were significant. These findings suggest and reinforce the complicated relationship between climate conditions in summer and spring in determining wildfire probabilities.

**Figure 4.**
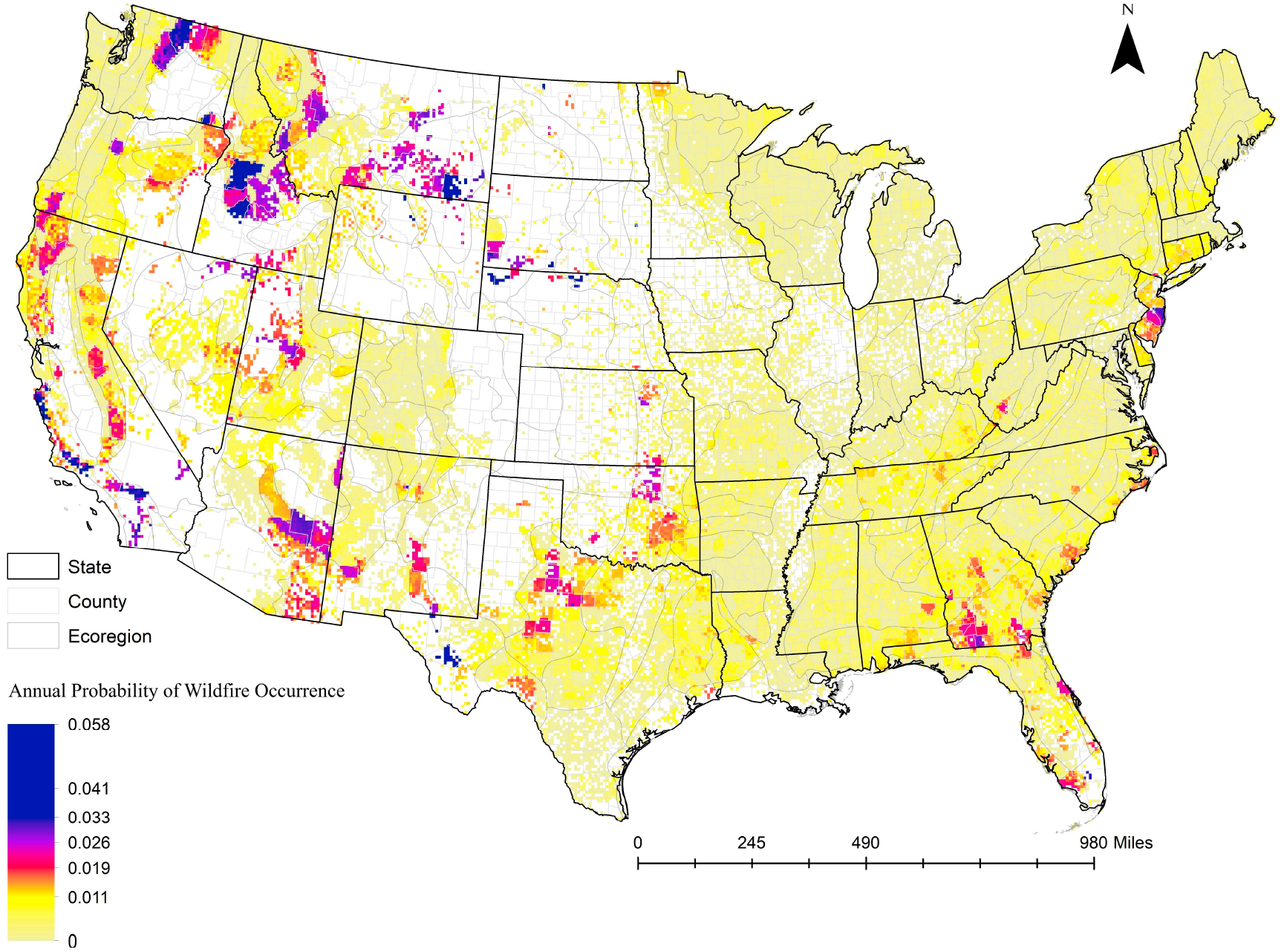
Annual probability of wildfire occurrences in FIA forest plots estimated using the fire risk equation across the continental United States. (Raster map download link: https://www.canr.msu.edu/socioeconomics/econ/data, accessed on 13 November 2022).

Figure 4 shows the annual probabilities of wildfire occurrences in the FIA forest plots using the fire risk model for the continental U.S. As expected and observed, our equation estimated higher wildfire probabilities in the west, especially in the California valleys, inner mountain regions between Cascade and the Rocky Mountains, and southwestern arid regions in California and Arizona. In the eastern U.S., some areas (mostly public forests) in Kentucky and Florida were more likely to burn. The dry regions of western Texas and Oklahoma were also more likely to burn than the eastern part of these states. Moreover, in the conterminous 48 States, our equation calibration showed that federally owned forests were more likely to burn than private forests. Fire suppression efforts in the past and reduced harvest in federal lands have played an important role in accumulating fuels for decades in the landscape escalating the wildfire risks [4].

### 3.2. Annual Probabilities of Biomass Loss Due to Tree Mortality from TreeBio-Loss Equation

The annual probability of tree mortality due to wildfire was negatively associated with summer precipitation and evapotranspiration and spring temperature (p<0.05), indicating higher mortality in the areas with lower precipitation and evapotranspiration during the fire season and with lower temperatures in the spring season (Table 4). The maximum summer temperature had no impact on tree mortality and was dictated mainly by moisture conditions. Spring precipitation and evapotranspiration also had no association with tree mortality, indicating moisture conditions in spring do not impact tree mortality due to wildfires.All quadratic relationships were significant (p<0.1), whereas the summer and spring temperatures were insignificant at 5%. This indicates that the marginal effect decreased with increasing summer precipitation, evapotranspiration, and spring temperature. However, the interaction between precipitation, temperature, and evapotranspiration in the fire and spring seasons were significant in the model, indicating a complex association between climatic conditions and tree mortality, making it difficult to establish a direct connection between one climatic parameter with the mortality.

**Figure 5.**
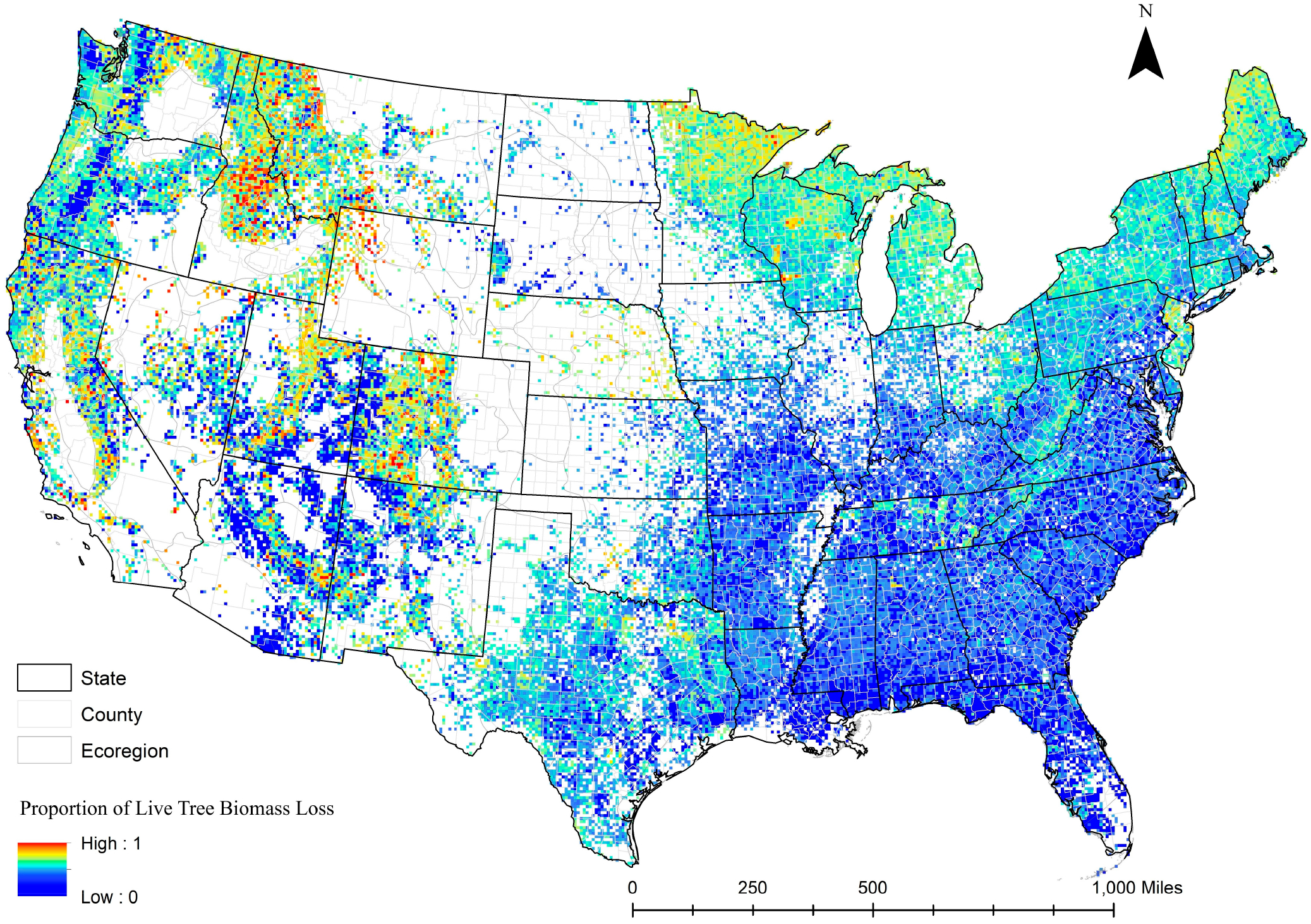
Proportion of live tree mortality in FIA forest plots predicted using TreeBio-Loss equation across the continental United States. (Raster map download link: https://www.canr.msu.edu/socioeconomics/econ/data, accessed on 13 November 2022).

Figure 5 shows the tree mortality estimates in the FIA forest plots using the TreeBio-Loss equation for the continental U.S. As expected, tree mortality was higher in the western U.S., where the wildfire probabilities were higher. The forests in the inner mountain region (Montana, Idaho, and Nevada) and inner California had a higher likelihood of tree mortality in the event of fire compared with other states. Tree mortality due to wildfire was very low in the southeast, likely because of the well-established practice of private ownership using monoculture and prescribed burns. In addition to climate variables, tree mortality was significantly lower in private forests and stand class with a higher age class. As expected, tree mortality was significantly higher in public forests compared with private forests because of less or no management as well as low firefighter presence and fire control efforts unless those lands are adjacent to the human settlements leading to higher burn severity and loss of biomass [24]. Moreover, tree mortality increased with the increase in available aboveground biomass. Old-growth forests have better chances of surviving because of their maturity and taller crowns and have fewer intermediate fuels to carry the surface fire to the crown [50]. The crown fire can be very intense and catastrophic.

### 3.3. Carbon Dioxide Emissions Due to the Wildfires in the United States

Results showed about 0.78 million hectares (ha) in AVG, 0.96 million hectares in ECP, and 1.43 million hectares of forests in the PLT scenario burned annually in the United States (Table 5). Compared with historical area burns (1.45 million hectares) from MTBS between 1985 and 2015 [3], the PLT scenarios produced the most accurate estimates with approximately 1% variability. Observing the historic wildfire-related tree mortality using FIA data, we estimate that stands affected by wildfires lose 23% of live trees annually across the continental U.S. due to wildfires. The CO_2_ emission estimates in *AVG* scenarios are low compared with the PLT scenarios, indicating that the current methods used for fire-related emissions in most forest sector models are underestimating the wildfire-related estimates. Our estimates using the fire risk and tree mortality equations at FIA plots estimated annual CO_2_ emissions of 6.10 million tons for PLT_*low*, 16.65 million tons for PLT_*med*, 22.75 million tons for PLT_*high* and 31.013 million tons for PLT_*cats* scenarios (Table 5). This clearly indicates that the increasing intensity of wildfires would significantly increase CO_2_ emissions. Lowering the intensity of fires can significantly lower the CO_2_ emissions from forests in the event of wildfire.

## 4. Discussion

The results show that maximum temperature and moisture conditions (precipitation and evapotranspiration) during the fire season and temperatures during spring influenced wildfire occurrences. A hot and dry summer coupled with a relatively cold spring aids in a higher probability of wildfires. Previous studies corroborate our findings in summer temperatures and moisture deficit significantly impacting the wildfire probabilities [8,21,26,37,65–67]. Westering et al. [8] concluded western fires result from warmer temperatures and longer dry fire seasons. Kerr et al. [21] concluded that the high-risk fire episodes are mainly related to the region’s temperature and precipitation, where precipitation’s role is smaller than temperatures, similar to our findings. Drever et al. [46] also concluded that lower precipitation increased fire occurrences and burned area. Siegel et al. [26] also concluded that fall (overlapping with our fire season) precipitation and temperatures were significant variables in estimating wildfire probabilities, including wind speed variables and PDSI. We did not include wind speed and PDSI in our model due to the lack of these data at FIA plots and to keep the model simple and parsimonious to use GCM climate data for future predictions on wildfire risk changes. However, we can argue that precipitation and evapotranspiration can proxy PDSI as both imply the moisture conditions at the plot, an important variable in wildfire risk estimation. Riley and Loehman [68] also concluded that increased burn probability was associated with lower fuel moisture and increased ignitions in hot and dry weather.

The relationship between spring climate on wildfire has not been directly assessed previously. Our study found a negative relationship between average spring maximum temperature and wildfire occurrences. Spring moisture conditions were not significant predictors of wildfire occurrences. However, Westerling et al. [8] found that earlier snowmelt dates correspond to increased wildfire frequency, increasing drought in summer. Starrs et al. [47] concluded that climate variables had little effect on vegetation type compared with ownership. This may be due to the extent of the study (geographic region), season selection (we use spring, not winter), and use of the non-linear model with interactions. Higher winter temperature helps to lose snow or moisture sooner; therefore, dry conditions in summer, whereas warmer springs (not the same as earlier snowmelt dates) help trees and shrubs to sprout earlier, hence more biomass or fuel loading for fire season. Coupled with dry fire season, this situation can significantly increase the occurrence and intensity of fires.

Tree mortality was associated with summer precipitation and evapotranspiration and weakly associated with spring temperatures, indicating mortality as a factor of moisture content. Tree mortality was not impacted by summer temperature and spring precipitation and temperatures. As expected, increased summer precipitation and evapotranspiration decreased tree mortality. Climate variables are generally used to predict fire intensity, which predicts tree mortality. This study estimates biomass loss due to tree mortality using climate variables and forest conditions with an FI of 57%, well in the acceptable range for such econometric analyses. As expected, plots with higher biomass but lower age stand experienced higher tree mortality.

On the other hand, significant quadratic relationships of climate variables with wildfire occurrences and tree mortality indicated a non-linear effect of climate on wildfires. The moisture conditions rather than the temperatures in the plots mostly dictated mortality. Although a direct impact of spring precipitation and evapotranspiration was not observed with wildfire occurrences and tree mortality, the interactions between precipitation, temperature, and evapotranspiration in the fire and spring seasons were significant in the model, indicating a complex association between climatic conditions and wildfire occurrences and tree mortality.

Landownerships were significant in determining wildfire probabilities and tree mortalities, with federal lands estimated to be more likely to have wildfires than private and state forest lands. The previous literature also showed the same relation [26,45,48, 68]. Siegel et al. [26] concluded that federal forests were 1.52 times more likely to burn in 2016, compared with 2.67 times in 1989, indicating decreasing yet significant differences in wildfire risk in federal and other lands. Starrs et al. [47] concluded that ownership, firefighting, and reserve statutes were detrimental to fire probabilities in California. In general, wildfires are not tackled until they reach Wildland Urban Interface (WUI). All previous studies concluded that fire risk is higher in the federally owned forest compared with others. Limited to no harvest as a management option in the federal lands has accumulated fuel. As expected, federally owned forests have a higher probability of wildfire and tree mortality than private and state lands. This result is correlated with firefighting practices and fuel management in federally owned forests where the fire is allowed to progress until it reaches WUI and, a minimal management is performed in federal forests allowing surface and ladder fuel development resulting in crown fires [27,47]. In addition, most of the federally owned forests are in the dry western part of the country.

We also present potential CO_2_ emissions due to wildfires at different scenarios of combustion factors. This study indicated other studies may have yielded lower estimations and projections of CO_2_ emissions due to wildfire using some traditional approaches, which generally use averages or county-level estimates of fire probabilities and tree mortality. Fire risk and tree mortality are estimated for a small geographical extent with a complex set of variables that could not be used for future projections. The approach developed in this study estimates the wildfire probabilities and biomass loss due to tree mortality at FIA plots with higher FI and lower RMSE compared with earlier statistical techniques, hence providing a finer resolution of these estimates at a national scale model.

This study has limitations. Notable limitations are the lack of fine-scale data to develop and run fire-related econometric models. After a fire, salvage harvest is possible and is not recorded in FIA, leading to an assumption that it was lost during the fire. This can increase the estimated biomass loss due to wildfire. FIA data are limited to fire-related variables to the presence or absence of fire between two measurements. FIA data do not report fire intensities, a key element in predicting mortality. The combustion conversion factor is not constant across the country but varies with fire intensity and severity at each plot. Since such data are lacking in FIA, we chose to use a constant combustion factor in this study. Further study should consider predicting fire intensities and combustion conversion factors at various intensities for a more precise estimation of wildfire risks.

## 5. Conclusions

This study developed fire risk and TreeBio-Loss equations for estimating wildfire probabilities and associated tree mortality using climate data for the U.S. and produced national maps for wildfire risk and fire-related tree mortality. These equations are a non-linear spatial autoregressive function of climatic conditions and forest attributes that provide a basis for estimating wildfire probability and associated tree mortality in FIA plots. Our study concludes that the fire season temperature and moisture conditions in an area significantly impact the wildfire probabilities, where the temperature had a positive effect and precipitation and evapotranspiration had a negative effect. Moreover, higher spring temperatures negatively affect wildfire probability by providing ideal conditions supporting biomass growth. On the other hand, tree mortality was mainly a function of moisture conditions in the fire season, with higher levels of moisture leading to lower tree mortality. Moreover, as found in many prior studies, federal lands were more likely to have wildfires than private and state forest lands.

Further, we use our estimates of national wildfire probabilities and tree mortality to evaluate how CO_2_ emissions from live trees vary in wildfires across a range of combustion scenarios. We also find that wildfire-related emissions are underestimated using previous methods under all combustion scenarios. For example, a technique using ten-year averages on fire and tree mortality would estimate 9.84 million tons of CO_2_ emissions, while our estimates using plot-level data showed a substantially higher emission level of 16.65 million tons of CO_2_. Future studies should incorporate these models of fire risk and tree mortality for better estimates of wildfire probability and biomass converted to CO_2_. We aim to apply these estimates in spatial equilibrium models of the forest sector, called the Land Use and Resource Allocation (LURA) model [47], to evaluate the efficacy of fuel reduction strategies. This would allow for the endogeneity of both future climate and biomass retention allowing a more robust suite of potential policy and climate scenarios informing the U.S. climate mitigation efforts.

## Figures and Tables

**Table 1.** Description and descriptive statistics of the climate and forest attributes of FIA forest plots in the continental United States.

Variable	Description	N^2^	Mean	SD^3^	Median	Min	Max
FIRE^1^	Number of plots experiencing wildfire and unknown fires between 2000 and 2015. 1 if a fire was reported, 0 otherwise.	150,350	0.07	0.25	0	0	1.00
TREELOSS^4^	Proportion of aboveground live biomass in dead or missing trees in remeasurements after a wildfire event in an FIA plot between 2000 and 2015.	4510	0.23	0.36	0.02	0	1.00
AGBIO	Total aboveground live tree biomass in a plot (1000 metric tons per hectare).	150,350	26.82	30.80	17.98	0	1049
AGE	Age of the forest stand of the plot.	150,350	65.24	55.40	57	0	1028
OWN	Ownership of the plot, 1 if federally owned, 0 otherwise.	150,350	0.25	0.44	0.00	0.00	1.00
Fire Season or Summer (July, August, September, and October)							
PPT	Average monthly precipitation in millimeters (mm) for the plot between 1985 and 2015.	150,350	82.00	33.13	90.99	2.59	246.60
TEMP	Average monthly maximum temperature for the plot in degree Celsius between 1985 and 2015.	150,350	25.09	4.52	24.95	10.48	41.95
ET	Average monthly evapotranspiration for the plot in mm between 1985 and 2015.	150,350	405	69	412	250	687
Spring Season (February, March, and April)							
PPTs	Average monthly precipitation in millimeters (mm) for the plot between 1985 and 2015.	150,350	87.28	50.41	85.21	5.24	594.57
TEMPs	Average monthly maximum temperature for the plot in degree Celsius between 1985 and 2015.	150,350	12.08	6.56	11.62	−2.00	27.98
ETs	Average monthly evapotranspiration for the plot in mm between 1985 and 2015.	150,350	220	72	217	95	492

1 Prescribed burn was not included;

2 total number of observations;

3 standard deviation;

4 only plots with fire were used to estimate tree mortality.

**Table 2.** Different scenarios to estimate CO_2_ emissions under different conversion rates and probability estimation methods in the continental United States.

			Rate of Conversion or Consumption	
Scenarios	Estimate of Wildfire Probability ($p_{i}$)	Estimate of Wildfire Probability ($m_{i}$)	Bole and Stump Biomass Combustion Rate ($f_{b}$)	Tops, Branches, and Sapling Biomass Combustion Rate ($f_{o}$)
AVG_*low*	$\overset{‾}{p}$ is the average (2005–2015) annual probability of wildfire occurrence for a county.	$\overset{‾}{m}$ is the average (2005–2015) annual probability of tree mortality for a county.	5%	50%
AVG_*med*			30%	80%
AVG_*high*			46%	92%
AVG_*cats*			80%	100%
ECP_*low*	${\overset{ˆ}{p}}_{c}$ is the annual probability of wildfire occurrence on c^th^ ecoregion within a county from the fire risk model.	${\overset{ˆ}{m}}_{i}$ is the annual probability of tree mortality on i^th^ plot from the TreeBio-Loss model.	5%	50%
ECP_*med*			30%	80%
ECP_*high*			46%	92%
ECP_*cats*			80%	100%
PLT_*low*	${\overset{ˆ}{p}}_{i}$ is the annual probability of wildfire occurrence on i^th^ plot with the fire risk model.	${\overset{ˆ}{m}}_{i}$ is the annual probability of tree mortality on i^th^ plot with the TreeBio-Loss model.	5%	50%
PLT_*med*			30%	80%
PLT_*high*			46%	92%
PLT_*cats*			80%	100%

**Table 3.** Fire risk model coefficients to estimate the wildfire probability in an ecoregion of a county of the continental United States.

Variables (n=3854)		Coefficient	Standard Error	p-Value
Intercept	$\overset{ˆ}{\alpha }$	−0.17030	0.07410	0.022
PPT	${\overset{ˆ}{\beta }}_{1}$	−0.00738	0.00070	0.000
TEMP	${\overset{ˆ}{\beta }}_{2}$	0.07472	0.02325	0.001
ET	${\overset{ˆ}{\beta }}_{3}$	−0.26480	0.07533	0.000
PPTs	${\overset{ˆ}{\beta }}_{4}$	−0.00011	0.00013	0.401
TEMPs	${\overset{ˆ}{\beta }}_{5}$	−0.08526	0.01618	0.000
ETs	${\overset{ˆ}{\beta }}_{6}$	4.74 × 10^−7^	4.07 × 10^−7^	0.244
PPT^2^	${\overset{ˆ}{\beta }}_{7}$	0.00001	2.25 × 10^−6^	0.000
TEMP^2^	${\overset{ˆ}{\beta }}_{8}$	−0.00556	0.00069	0.000
ET^2^	${\overset{ˆ}{\beta }}_{9}$	−0.05155	0.01604	0.001
PPTs^2^	${\overset{ˆ}{\beta }}_{10}$	−0.00304	0.00046	0.000
TEMPs^2^	${\overset{ˆ}{\beta }}_{11}$	2.47 × 10^−6^	7.33 × 10^−7^	0.001
ETs^2^	${\overset{ˆ}{\beta }}_{12}$	2.80 × 10^−12^	9.47 × 10^−13^	0.003
PPT * TEMP	${\overset{ˆ}{\beta }}_{13}$	−0.00004	0.00005	0.413
PPT * ET	${\overset{ˆ}{\beta }}_{14}$	0.00160	0.00031	0.000
PPT * PPTs	${\overset{ˆ}{\beta }}_{15}$	−0.00001	2.02 × 10^−6^	0.000
TEMP * TEMPs	${\overset{ˆ}{\beta }}_{16}$	0.00644	0.00107	0.000
TEMP * ET	${\overset{ˆ}{\beta }}_{17}$	0.02710	0.00363	0.000
ET * ETs	${\overset{ˆ}{\beta }}_{18}$	−2.65 × 10^−7^	8.39 × 10^−8^	0.002
OWN	${\overset{ˆ}{\delta }}_{1}$	0.01358	0.00292	0.000
Spatial autocorrelation	ς	1.33100	0.00934	0.000
Root mean squared error (RMSE) *		0.077		
Fit index (FI) **		0.56		

* Root mean squared error is the standard deviation of the residuals (prediction errors).

** Fit index, similar to r-squared in linear regressions, tell how well-observed data fit a particular probability distribution.

**Table 4.** TreeBio-Loss model to estimate biomass loss due to tree mortality in the forested FIA plots of the continental United States.

Variables (n=4510)		Coefficient	Standard Error	p-Value
Intercept	$\overset{ˆ}{\alpha }$	2.26700	0.44450	0.000
PPT	${\overset{ˆ}{\beta }}_{1}$	−0.00513	0.00213	0.016
TEMP	${\overset{ˆ}{\beta }}_{2}$	0.10640	0.07137	0.136
ET	${\overset{ˆ}{\beta }}_{3}$	−2.51700	0.60840	0.000
PPTs	${\overset{ˆ}{\beta }}_{4}$	0.00038	0.00038	0.315
TEMPs	${\overset{ˆ}{\beta }}_{5}$	−0.12660	0.05568	0.023
ETs	${\overset{ˆ}{\beta }}_{6}$	1.00300	0.62190	0.107
PPT^2^	${\overset{ˆ}{\beta }}_{7}$	0.00001	0.00000	0.003
TEMP^2^	${\overset{ˆ}{\beta }}_{8}$	−0.00418	0.00214	0.050
ET^2^	${\overset{ˆ}{\beta }}_{9}$	0.54960	0.13870	0.000
PPTs^2^	${\overset{ˆ}{\beta }}_{10}$	3.85 × 10^−6^	1.13 × 10^−6^	0.001
TEMPs^2^	${\overset{ˆ}{\beta }}_{11}$	−0.00295	0.00163	0.070
ETs^2^	${\overset{ˆ}{\beta }}_{12}$	0.36410	0.15080	0.015
PPT * TEMP	${\overset{ˆ}{\beta }}_{13}$	−0.00032	0.00009	0.001
PPT * ET	${\overset{ˆ}{\beta }}_{14}$	0.00267	0.00077	0.001
PPT * PPTs	${\overset{ˆ}{\beta }}_{15}$	−0.00002	4.00 × 10^−6^	0.000
TEMP * TEMPs	${\overset{ˆ}{\beta }}_{16}$	0.00778	0.00347	0.025
TEMP * ET	${\overset{ˆ}{\beta }}_{17}$	0.00128	0.00806	0.874
ET * ETs	${\overset{ˆ}{\beta }}_{18}$	−0.76150	0.26940	0.005
OWN	${\overset{ˆ}{\delta }}_{1}$	0.05385	0.00843	0.000
AGE	${\overset{ˆ}{\delta }}_{2}$	−0.00245	0.00007	0.000
AGBIO	${\overset{ˆ}{\delta }}_{3}$	3.49 × 10^−7^	9.74 × 10^−8^	0.000
Spatial autocorrelation	ρ	0.42470	0.02871	0.000
Root mean squared error (RMSE) *		0.237		
Fit index (FI) **		0.57		

* Root mean squared error is the standard deviation of the residuals (prediction errors).

** Fit index, similar to r-squared in linear regressions, tell how well-observed data fit a particular probability distribution.

**Table 5.** Annual estimates of forest area burned, live biomass lost, and the CO_2_ emissions under different combustion rates and various scenarios due to wildfires in the continental United States.

Scenarios	Scenario Description	Wildfire Burn Area (1000 ha) A	Total AG Biomass in the Burn Area (MMt)	Live Tree Biomass Loss with Mortality (MMt) B	CO_2_ Emissions (MMt/year) C
AVG_*low*	$\begin{array}{c} p_{i}=\overset{‾}{p},m_{i}=\overset{‾}{m}\\ f_{b}=0.05,f_{o}=0.5 \end{array}$	775.18	55.43	2.04	3.73
AVG_*med*	$\begin{array}{c} p_{i}=\overset{‾}{p},m_{i}=\overset{‾}{m}\\ f_{b}=0.3,f_{o}=0.8 \end{array}$	775.18	55.43	5.37	9.84
AVG_*high*	$\begin{array}{c} p_{i}=\overset{‾}{p},m_{i}=\overset{‾}{m}\\ f_{b}=0.46,f_{o}=0.92 \end{array}$	775.18	55.43	7.28	13.35
AVG_*cats*	$\begin{array}{rr} & p_{i}=\overset{‾}{p},m_{i}=\overset{‾}{m}\\ & f_{b}=0.8,f_{o}=1 \end{array}$	775.18	55.43	10.79	19.79
ECP_*low*	$\begin{array}{c} p_{i}={\overset{ˆ}{p}}_{c},m_{i}={\overset{ˆ}{m}}_{i}\\ f_{b}=0.05,f_{o}=0.5 \end{array}$	964.61	77.44	2.53	4.63
ECP_*med*	$\begin{array}{rr} & p_{i}={\overset{ˆ}{p}}_{c},m_{i}={\overset{ˆ}{m}}_{i}\\ & f_{b}=0.3,f_{o}=0.8 \end{array}$	964.61	77.44	7.13	13.07
ECP_*high*	$\begin{array}{c} p_{i}={\overset{ˆ}{p}}_{c},m_{i}={\overset{ˆ}{m}}_{i}\\ f_{b}=0.46,f_{o}=0.92 \end{array}$	964.61	77.44	9.81	17.99
ECP_*cats*	$\begin{array}{c} p_{i}={\overset{ˆ}{p}}_{c},m_{i}=\overset{‾}{m}\\ f_{b}=0.8,f_{o}=1 \end{array}$	964.61	77.44	4.88	27.28
PLT_*low*	$\begin{array}{c} p_{i}={\overset{ˆ}{p}}_{i,},m_{i}={\overset{ˆ}{m}}_{i}\\ f_{b}=0.05,f_{o}=0.5 \end{array}$	1426.11	115.61	3.33	6.10
PLT_*med*	$\begin{array}{rr} & p_{i}={\overset{ˆ}{p}}_{i},m_{i}={\overset{ˆ}{m}}_{i}\\ & f_{b}=0.3,f_{o}=0.8 \end{array}$	1426.11	115.61	9.08	16.65
PLT_*high*	$\begin{array}{c} p_{i}={\overset{ˆ}{p}}_{i},m_{i}={\overset{ˆ}{m}}_{i}\\ f_{b}=0.46,f_{o}=0.92 \end{array}$	1426.11	115.61	12.41	22.75
PLT_*cats*	$\begin{array}{c} p_{i}={\overset{ˆ}{p}}_{i},m_{i}=\overset{‾}{m}\\ f_{b}=0.8,f_{o}=1 \end{array}$	1426.11	115.61	18.61	34.13

$\overset{‾}{p}$ is the average annual probability of wildfire occurrence for fire events between 2005 and 2015 for each county. ${\overset{ˆ}{p}}_{i}$ is the annual probability of wildfire occurrence on i^th^ plot with the fire risk model. ${\overset{ˆ}{p}}_{c}$ is the annual probability of wildfire occurrence on $c^{\text{th}}$ ecoregion within a county with the fire risk model. $\overset{‾}{m}$ is the 10-year average tree mortality for the U.S. ${\overset{ˆ}{m}}_{i}$ is the annual probability of tree mortality on i^th^ plot with the TreeBio-Loss model. $f_{b}$ is the rate of combustion or consumption of bole and stump biomass by wildfire to convert it into CO_2_. $f_{0}$ is the rate of combustion or consumption of tops, branches, and sapling biomass by wildfire to convert it into CO_2_.

## Data Availability

The raster maps for fire risk and TreeBio-Loss are available from Forest Economics Lab at Michigan State University (Raster map download link: https://www.canr.msu.edu/socioeconomics/econ/data, accessed on 13 November 2022). Or please get in touch with Raju Pokharel (raju2020@msu.du) to get maps.
